# Clinical Predictors and Pathogen Resistance Dynamics in Hospitalized Patients with Urinary Tract Infections: A 2025 Institutional Study

**DOI:** 10.3390/microorganisms14040916

**Published:** 2026-04-18

**Authors:** Ruxandra Laza, Ioana-Melinda Luput-Andrica, Adelina-Raluca Marinescu, Talida-Georgiana Cut, Alexandra Herlo, Andra-Elena Saizu, Andreea-Cristina Floruncut, Narcisa Nicolescu, Romanita Jumanca, Daniela-Ica Rosoha, Voichita Elena Lazureanu, Romosan Ana-Maria

**Affiliations:** 1 Department XIII, Discipline of Infectious Diseases, “Victor Babes” University of Medicine and Pharmacy Timisoara, E. Murgu Square, Nr. 2, 300041 Timisoara, Romania; laza.ruxandra@umft.ro (R.L.); adelina.marinescu@umft.ro (A.-R.M.); talida.cut@umft.ro (T.-G.C.); alexandra.mocanu@umft.ro (A.H.); nicolescu.narcisa@umft.ro (N.N.); lazureanu.voichita@umft.ro (V.E.L.); 2 Doctoral School, “Victor Babes” University of Medicine and Pharmacy Timisoara, E. Murgu Square, Nr. 2, 300041 Timisoara, Romania; andra.saizu@umft.ro (A.-E.S.); daniela.rosoha@umft.ro (D.-I.R.); 3 Center for Ethics in Human Genetic Identification, “Victor Babes” University of Medicine and Pharmacy Timisoara, E. Murgu Square, Nr. 2, 300041 Timisoara, Romania; 4 Romanian and Foreign Languages Department, “Victor Babes” University of Medicine and Pharmacy Timisoara, 300041 Timisoara, Romania; romanita.jumanca@umft.ro; 5 Psihiatry, Neurosciences Department, “Victor Babes” University of Medicine and Pharmacy Timisoara, E. Murgu Square, Nr. 2, 300041 Timisoara, Romania; romosan.ana@umft.ro

**Keywords:** Gram-negative bacteria, catheter-associated urinary tract infections, urinary tract infections, antimicrobial resistance, multidrug resistance, cephalosporins, *E. coli*, *Klebsiella pneumoniae*, fosfomycin

## Abstract

The escalating prevalence of antimicrobial resistance (AMR) in Gram-negative uropathogens represents a critical bottleneck in global clinical management. This study evaluated shifting resistance phenotypes and patient risk profiles to identify independent predictors of multidrug resistance (MDR). A comprehensive retrospective analysis was conducted on a cohort of 318 patients, utilizing statistical modeling to evaluate the impact of demographics, prolonged hospitalization, and comorbidities on MDR. Findings revealed a significant longitudinal exacerbation of resistance since 2012. A majority of *Klebsiella pneumoniae* strains and nearly all *Myroides* and *Providencia* species exhibited high-level resistance to cephalosporin/beta-lactamase inhibitor combinations. While high-dose piperacillin-tazobactam remains a therapeutic alternative, its utility is increasingly constrained by escalating Minimum Inhibitory Concentrations (MICs) for *Klebsiella* and *Escherichia coli* (*E. coli*). Statistical modeling identified advanced age as the primary independent driver, with MDR risk increasing linearly with every additional year of age. Furthermore, indwelling catheterization was strongly associated with resistant infections, while human immunodeficiency virus (HIV) status emerged as a significant cofactor in the selection of highly resistant strains. These findings underscore the need for a critical recalibration of therapeutic frameworks, prioritizing precision-guided stewardship. Pharmacodynamic optimization, through extended or continuous infusion regimens and individualized loading doses, is essential to mitigate the clinical burden of resistant pathogens within vulnerable geriatric cohorts.

## 1. Introduction

Urinary tract infections (UTIs) represent a major health burden, with their incidence escalating from 5294.5 to 8560.6 per 100,000 individuals over the last two decades. Notably, mortality rates associated with these infections have surged by 190% since 1990 [1], transforming UTIs from common clinical ailments into critical drivers of systemic sepsis. Beyond their high prevalence, the pathogenesis of these infections is increasingly understood through the gut-skin-urinary axis—a complex transmission route where the gastrointestinal tract and cutaneous flora act as primary reservoirs for uropathogens [2]. This bacterial translocation from the commensal flora is further amplified by selective pressure from prior antibiotic use, which shifts commensal flora toward highly resistant opportunistic phenotypes. In hospitalized patients, this axis is facilitated by indwelling urinary catheters [3], which promote the migration of colonizing bacteria from the skin and gut reservoirs into the bladder. This process leads to the formation of recalcitrant polymicrobial biofilms, increasing the risk of infection by 3% to 7% for each day of catheterization [4].

The clinical management of UTIs is profoundly complicated by Gram-negative bacteria (GNB) [5,6,7,8], which are the predominant etiological agents in both community-acquired and nosocomial settings [8]. Species such as *Klebsiella pneumoniae*, *Pseudomonas aeruginosa*, and *Acinetobacter baumannii* pose a critical hospital-wide challenge due to their extensive multidrug-resistant (MDR) profiles [9]. The proliferation of extended-spectrum beta-lactamase (ESBL)-producing strains, particularly those carrying the blaCTX-M genotypes, has necessitated the use of carbapenems as first-line empirical therapy; however, the worldwide emergence of carbapenemase-producing uropathogens (e.g., KPC-, NDM-, and OXA-48 producing isolates) now jeopardizes these “last-line” agents. Consequently, UTIs of Gram-negative etiology have become a focal point of antimicrobial stewardship, as high resistance levels and inadequate drug penetration into renal and bladder tissues (pharmacokinetic/pharmacodynamic failures) often lead to diminished therapeutic responses [10,11,12,13].

In this landscape of rapidly evolving resistance, the antibiogram is no longer merely a supportive tool but an essential clinical and ethical mandate for the verification and refinement of empirical therapy. Given that approximately 50% of bloodstream infections secondary to carbapenem-resistant *Enterobacteriaceae* result in mortality, relying solely on empirical protocols without timely microbiological confirmation risks treatment failure, urosepsis, and the escalation of healthcare costs [14,15].

While global epidemiological shifts are well-documented, there remains a critical need to determine whether these resistance patterns are universal or specific to localized hospital environments and are influenced by distinct prescribing patterns and patient demographics [13,14,15].

This study addresses this gap by evaluating contemporary epidemiology and temporal shifts in GNB resistance at our institution. Previously, internal surveillance conducted between 2000 and 2010 revealed a substantial decline in fluoroquinolone susceptibility, from 88% to 62%, alongside 20% of isolates exhibiting resistance to cephalosporins. Although these longitudinal findings were not previously published in a peer-reviewed journal, they were presented locally to identify necessary improvements and to implement targeted antimicrobial stewardship measures within our hospital. Building on those findings, the current study aims to evaluate modern resistance patterns, identify temporal shifts in antimicrobial susceptibility, and characterize the risk factors predisposing patients to GNB-related healthcare-associated infections. By providing a localized model of how targeted stewardship can address the escalating crisis of MDR uropathogens, this article offers essential data for precision-based prescribing in the face of the global antimicrobial resistance (AMR) crisis.

## 2. Materials and Methods

### 2.1. Study Setting and Data Collection

This retrospective, observational study was conducted at the “Victor Babeș” Clinical Hospital for Infectious Diseases and Pulmonology in Timișoara. The study followed the Strengthening the Reporting of Observational Studies in Epidemiology (STROBE) guidelines to ensure methodological transparency. Clinical and microbiological data were extracted from five specialized departments: Infectious Diseases, Pulmonology, Respiratory Rehabilitation, Thoracic Surgery, and Intensive Care. A total of 318 non-duplicate, positive urine cultures were analyzed from 1 January 2025 to 30 November 2025. To ensure that the microbiological isolates reflected the patients’ status at the time of admission and to strictly exclude nosocomial acquisitions, all clinical specimens were collected cross-sectionally within the first 48 h of their hospital stay. To prevent data redundancy, in instances where multiple antimicrobial susceptibility tests (ASTs) were performed for the same bacterial phenotype in a single patient within a 30-day window, only the initial isolate was included in the final analysis.

Strict exclusion criteria were implemented to minimize confounding variables. To establish a clear baseline of uropathogenic resistance in the general population, patients with anatomical abnormalities of the urinary tract, or a solitary functional kidney (congenital or surgical) were excluded. Patients with chronic kidney disease (CKD) were also omitted; this was a deliberate decision to avoid bias, as CKD patients often present with distinct resistance profiles and altered renal clearance could skew the general uropathogenic baseline. Furthermore, pregnant women and individuals who had received antibiotic therapy within the preceding 3–6 months were excluded to eliminate the influence of recent selective pressure. Finally, patients unable to provide informed consent and those with polymicrobial isolates that did not meet specific clinical criteria were excluded to maintain interpretive clarity.

Significant bacteriuria was defined as ≥10^5^ CFU/mL with concomitant leukocyturia (>10^4^ leukocytes/mL), according to national guidelines [16]. Bacterial identification and antimicrobial susceptibility tests (ASTs) were conducted using automated systems and interpreted based on the 2025 European Committee on Antimicrobial Susceptibility Testing (EUCAST) breakpoints. Resistance was assessed against a panel that included amoxicillin/clavulanic acid, nitrofurantoin, trimethoprim/sulfamethoxazole, nalidixic acid, ofloxacin, ciprofloxacin, and ceftriaxone. Extended-Spectrum Beta-Lactamase (ESBL) production was verified using the double-disc synergy test. MDR was defined according to the joint ECDC/CDC (Centers for Disease Control and Prevention) consensus as acquired non-susceptibility to at least one agent in three or more antimicrobial categories routinely used for treatment [17,18]. To provide a comprehensive clinical landscape, *Enterococcus* spp. were integrated into the analysis due to their role as critical opportunistic pathogens under the selective pressure of broad-spectrum cephalosporins [19].

Data extraction prioritized a comprehensive analysis of demographic and clinical variables. Gender analysis was a focal point of the study, with the cohort comprising 207 women and 111 men. Given that resistance profiles in pediatric patients differ significantly from those in the geriatric population due to varying comorbidity burdens, the data were stratified by age. The cohort included five patients under the age of 18, while the majority of the study population was concentrated in the elderly age groups: 112 patients aged 71–80 years and 68 patients aged 61–70 years. Additional variables included place of origin (rural vs. urban), the presence of an indwelling urinary catheter (IUC), and the total length of hospitalization (LOH). Microbiological variables focused on the identification of *Escherichia coli* (*E. coli*), *Proteus mirabilis* (*P. mirabilis*), and *Klebsiella pneumoniae* (*K. pneumoniae*).

### 2.2. Data Analysis

Statistical analysis was performed using IBM SPSS Statistics software (version 20.0, IBM Corp., Armonk, NY, USA). The normality of numerical data was assessed via Shapiro–Wilk and Kolmogorov–Smirnov tests; given the non-Gaussian distribution (*p* < 0.05), non-parametric tests were employed. The Mann–Whitney U test was used for comparisons between two independent groups, while the Kruskal–Wallis test was used for comparisons among three or more groups, followed by Dunn’s post hoc analysis with Bonferroni correction.

Categorical variables were evaluated using the *X*^2^ (*Chi-square*) test, which allowed for the examination of associations between categories. Additionally, correlations among variables were determined using Spearman’s rho.

A binary logistic regression model was developed to identify independent predictors of MDR status, with model fit assessed via the Hosmer-Lemeshow test. Results are reported as Odds Ratios (ORs) with 95% confidence intervals (CIs). The threshold for statistical significance was established at *p* < 0.05 [20].

### 2.3. Ethical Considerations

The study protocol was reviewed and approved by the Ethics Committee of the “Victor Babeș” Clinical Hospital of Infectious Diseases and Pneumophysiology, Timișoara (Approval No. 12334). Although the study covers a period ending in November 2025, the final formal approval was issued on 30 December 2025, specifically to authorize the retrospective analysis of the existing clinical data in accordance with ethical standards for human research. All participants (or legal guardians for minors) had previously provided written informed consent at the time of admission for their data to be used for clinical and research purposes [21].

## 3. Results

### 3.1. Distribution and Prevalence of Bacterial Species Isolated from Urine Cultures

**Figure 1:**
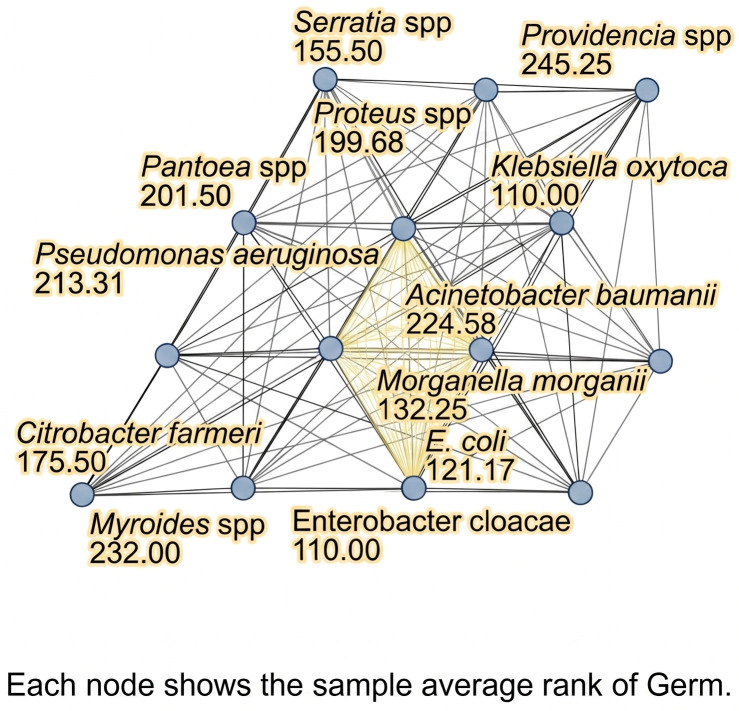
Distribution and prevalence of bacterial species isolated from urine cultures. The network diagram illustrates the statistical difference in the distribution and prevalence of identified uropathogens. Each node represents a bacterial species, with a numerical value indicating its sample average rank based on isolation frequency. Yellow lines denote pairs of pathogens with statistically significant differences in their distribution patterns. Black lines indicate species with similar epidemiological distributions across the hospital settings.

As illustrated in Figure 1 and Table 1, we investigated the distribution of bacterial isolates from urinary cultures, providing a comprehensive overview of the uropathogens associated with UTIs. The findings revealed a predominance of *E. coli*, which was identified in 143 (44.9%) isolates. Notably, 129 (90.2%) of these strains originated from the Infectious Diseases Department (IDD), while no cases were reported in the Intensive Care Unit (ICU).

In contrast, *K. pneumoniae* showed high environmental adaptability, with a significant presence in both the IDD (*n* = 77) and ICU (*n* = 18).

High-risk pathogens such as *Acinetobacter* species (*Acinetobacter* spp.) were detected exclusively within the ICU (six positive samples), confirming their association with critical care settings. Additionally, *Enterobacter cloacae* was identified in a single isolate from the IDD, indicating a comparatively low incidence relative to other GNB.

*Citrobacter freundii* was isolated from one sample in the IDD and another in the Pulmonology Department (PD), suggesting the potential dissemination of this bacterium between different specialized treatment units.

*Morganella morganii* was identified in two samples from patients hospitalized in the IDD, indicating a low prevalence within the cohort. Conversely, *Myroides* spp. were detected in one patient in the ICU and two in the IDD, emphasizing the necessity of monitoring these rare but clinically significant pathogens in the context of UTIs.

The genus *Proteus* was detected with greater frequency in the IDD (9 of 11 cases), compared to single isolates from the ICU and the Respiratory Rehabilitation Department (RRD), suggesting a diverse range of infection sources. *Providencia* spp. were found exclusively in the IDD with six positive isolates, whereas *Klebsiella oxytoca* (*K. oxytoca*) was identified in only a single isolate within the same department.

*Pseudomonas aeruginosa* was identified in 13 samples, with 10 originating from the IDD, 2 from the Pulmonology Department, and one from the ICU. This distribution confirms a significant prevalence of *Pseudomonas* in the UTIs, highlighting its potential to contribute to severe clinical outcomes.

*Serratia* was isolated exclusively from the IDD in two distinct isolates, while *Pantoea* spp. were noted in a single isolate from the same department. Although less common, these species contribute to the epidemiological complexity of UTIs within the clinical environment.

Although the primary objective of this study was to characterize the resistance profiles of GNB, *Enterococcus* spp. (a Gram-positive genus) were included in the final analysis to provide a comprehensive representation of the local uropathogenic landscape. This decision was based on the high clinical prevalence of *Enterococcus* identified during the study period (5% of the total isolates) and its significant role in the etiology of healthcare-associated UTIs. In clinical practice, *Enterococcus* often emerges as a critical pathogen under the selective pressure of broad-spectrum cephalosporins—agents frequently used to target GNB [22]. By including this genus, the study shifts from a strictly taxonomic focus on GNB to a more clinical, syndromic approach, reflecting the actual microbial challenges faced by clinicians in the Infectious Diseases and Intensive Care settings. This enables a more nuanced evaluation of the co-existing and competitive dynamics between Gram-negative and Gram-positive uropathogens within hospital environments [23].

### 3.2. Baseline Demographic and Clinical Characteristics of Patients

In the present study, a comprehensive analysis was conducted on a cohort of 318 patients receiving care across five distinct hospital departments. Within this group, 111 men (34.9%) and 207 women (65.1%) were identified. The mean age of the study population was 66.66 ± 17.41 years, with an age range spanning from 1 to 96 years, reflecting considerable demographic diversity.

**Figure 2:**
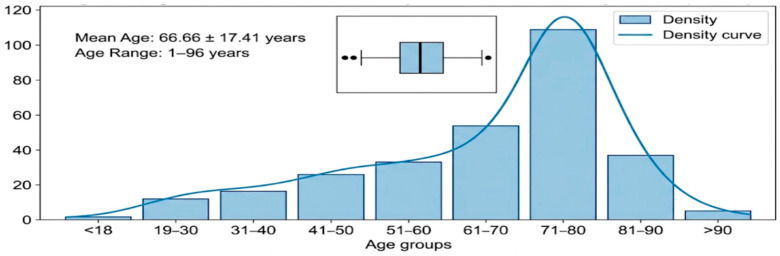
Frequency Distribution of Patient Age by Decade. Distribution of patients across age groups showing a clear shift toward the geriatric population.

As shown in Figure 2, age-specific analysis demonstrated a marked predominance of cases among older adults. The pediatric group (<18 years) comprised 5 patients (1.57%; four females and one male). Young and middle-aged adults were distributed as follows: 11 patients aged 19–30 years (3.46%; six females, five males), 19 patients aged 31–40 years (5.97%; 14 females, five males), 11 patients aged 41–50 years (3.46%; eight females, three males), and 33 patients aged 51–60 years (10.38%; 21 females, 12 males). The elderly cohorts represented the largest proportion of the study population, with 68 patients aged 61–70 years (21.38%; 32 females, 36 males), and 112 patients in the 71–80-year interval (35.22%; 76 females, 36 males). Furthermore, the “oldest-old” category included 56 patients aged 81–90 years (17.61%; 43 females, 13 males) and three patients exceeding 90 years of age (0.94%; 100% females).

Notably, 128 individuals (40.3%) originated from rural areas, whereas 190 (59.7%) resided in urban settings. This distribution may reflect differing healthcare-seeking behaviors; patients from rural regions might prioritize proximity to local facilities to avoid travel constraints associated with our center, particularly for mild symptoms. The majority of the study population was managed within the IDD, representing 77.99% of the whole cohort (*n* = 248). Additionally, both the pulmonology and ICU departments recorded significant numbers of positive urine cultures, with 36 (11.31%) and 27 (8.5%) patients, respectively.

In a similar yet noteworthy proportion, the Respiratory Rehabilitation Department reported six patients with positive urine cultures (1.88%). Conversely, the Thoracic Surgery Department identified only one patient with a UTI, corresponding to 0.31% of the overall study population.

The Mann–Whitney *U* test indicated significant differences between male and female patients concerning both average age and the length of hospitalization (LOH). Specifically, the mean age of female patients (67.27 years) exceeded that of male patients (65.52 years). Interestingly, despite their younger average age, men had a significantly longer LOH compared with women, reaching strong statistical significance (*p* < 0.0001; *U* = 8456; *Z* = 3.88). On average, male patients were hospitalized for 16.44 days, while female patients had a mean duration of 9.66 days.

Regarding the place of origin, the analysis revealed no statistically significant differences between patients from rural and urban areas in relation to age or LOH. These findings suggest that while demographic and hospitalization variations exist between the sexes, geographical factors do not appear to significantly impact these clinical outcomes.

The average LOH for the entire cohort was 13.27 days (range 1–69 days), reflecting the variability of cases and the complexity of required treatments. Among the evaluated samples, 181 (56.9%) demonstrated MDR status, while 137 samples (43.1%) showed no resistance to multiple antibiotic classes.

In the statistical analysis, no significant correlation was found between the specific pathogens identified and the patients’ average age (*p* = 0.26). However, the Kruskal–Wallis test indicated significant differences related to the LOH. Utilizing the post hoc Dunn-Bonferroni procedure, we noted significant variations in the mean hospitalization duration for patients in whom *K. pneumoniae*, *Pseudomonas aeruginosa* and *Acinetobacter baumannii* were identified, compared to those in whom *E. coli* was detected.

The findings revealed that the mean of LOH for patients with *K. pneumoniae* was 14.51 days (*p* < 0.0001 relative to *E. coli*), while those with *P. aeruginosa* had an average stay of 13.61 days, which did not reach statistical significance (*p* = 0.94). Notably, patients with *Acinetobacter baumannii* required an average of 54.83 days (*p* = 0.05). In stark contrast, patients with *E. coli* experienced a significantly shorter average hospitalization of only 7.86 days. These results suggest that infections caused by highly virulent or resistant organisms result in increased clinical severity, leading to extended hospitalization for treatment and recovery.

**Figure 3:**
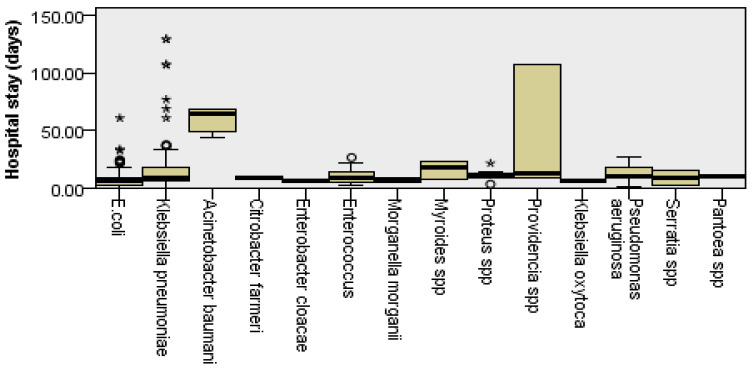
Length of hospital stay by Pathogen. The boxplot illustrates the distribution of hospital stay duration across the identified bacterial isolates. The central horizontal line within each box represents a median LOH, while the box boundaries indicate the interquartile range. Whiskers represent the spread of the data, and outliners are depicted as individual points (o and *). A stark contrast is observed between the brief clinical course of *E. coli* infections and the prolonged hospitalization required for high-risk pathogens such as *Acinetobacter baumannii* and *Providencia* spp.

Moreover, the identified pathogens exhibited significant differences in average LOH, suggesting that specific high-risk pathogens play a crucial role in increasing the severity and management complexity of urinary infections. The detailed data distribution is depicted in Figure 3.

### 3.3. Evaluating Multi-Resistance Patterns in Relation to Clinical Settings and Patient Demographic

MDR to antibiotics was observed in 181 patients, accounting for 56.9% of the whole cohort. Within this subgroup, 74 (40.9%) were male and 107 (59.1%) were female. Statistical analysis utilizing the *chi-square* *(X*^2^*)* test indicated that MDR status was significantly more prevalent among male patients (*p* = 0.01). Specifically, isolates from 74 out of 111 men exhibited MDR, representing a proportion of 66.7%, while 37 (33.3%) did not. In contrast, 107 out of 207 women (51.7%) were identified as having MDR isolates, compared to 100 (48.3%) who presented with non-MDR strains. These findings suggest a significant sex-based disparity in the distribution of antimicrobial resistance.

Regarding the place of residence, the analysis did not indicate statistically significant differences in the frequency of MDR isolates between patients from urban and rural settings, suggesting that geographical origin was not a primary driver of resistance in this cohort. Among the 128 patients from rural areas, 65 (50.8%) presented with MDR isolates, while 63 (49.2%) did not. For the 190 patients from urban settings, a numerically stronger trend was noted, with 116 patients (61.1%) exhibiting MDR isolates compared to 74 (38.9%) with strains without resistance. Despite the higher proportion in the urban areas, the lack of statistical significance implies that the risk of encountering MDR pathogens remains relatively consistent across both residential environments.

The *chi-square* test revealed a significantly higher prevalence of MDR strains among patients admitted to specific clinical departments (*p* < 0.001). In the ICU, 100% of the identified pathogens demonstrated an MDR phenotype. Furthermore, in the Respiratory Rehabilitation Department, five out of six patients (83.3%) presented with antibiogram results positive for MDR-positive isolates.

In the Pulmonology Department, 66.67% of patients (24 out of 36) were diagnosed with MDR uropathogens. In contrast, within the IDD, the proportion of patients exhibiting MDR strains was comparable to those with susceptible strains, with 50.40% (125 out of 248) of evaluated patients presenting with MDR isolates.

The LOH was significantly greater for patients infected with MDR organisms (*p* < 0.0001; *U* = 7827; *Z* = −5.64). This correlation underscores the necessity for rigorous monitoring and more comprehensive medical interventions in these cases. These data emphasize the critical nature of antimicrobial resistance across various clinical departments and its direct impact on healthcare resource utilization.

### 3.4. Multidrug Resistance Patterns Among Uropathogens

Statistical analysis utilizing the *Chi-square* (*X*^2^) test revealed that MDR was significantly more prevalent in infections caused by specific pathogens: *K. pneumoniae* (98/111, 88.3%), *Acinetobacter baunannii* (6/6, 100%), *Enterobacter cloacae* (1/1, 100%), *Morganella morganii* (2/2, 100%), *Myroides* spp. (3/3, 100%), *Providencia* spp. (5/6, 83.3%), *Pseudomonas aeruginosa* (8/13, 61.5%), and *Pantoea* spp. (1/1, 100%).

**Figure 4:**
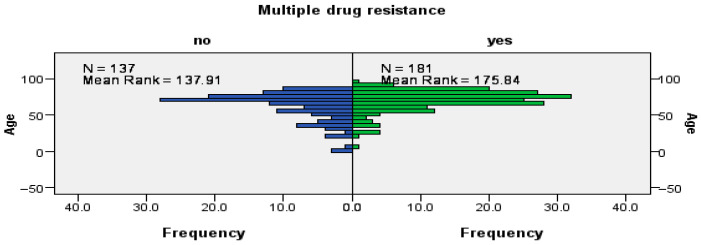
Average Length of Hospitalization for Patients with MDR strains. The population pyramids compare the age frequency between patients with susceptible isolates (blue, *n* = 137) and those with MDR strains (green, *n* = 181). The higher Mean Rank observed in the MDR group compared to the non-MDR group visually confirms that advanced age is significantly associated with the presence of the multidrug-resistant uropathogens. This distribution underscores the increasing clinical complexity and the higher probability of resistant infections in the geriatric population.

The mean age of patients with MDR strains was 70.03 ± 14.73 years, whereas the mean age for patients with non-MDR strains was 62.2 ± 19.6 years. The Mann–Whitney U test demonstrated a significant age disparity between the two groups (*U* = 9440.5; *Z* = −3.65; *p* < 0.0001), indicating that patients harboring MDR isolates are significantly older. These findings suggest a robust association between specific uropathogen species, advanced age, and the prevalence of antimicrobial resistance, as illustrated in Figure 4.

The average LOH for patients with MDR strains was 17.32 ± 22.6 days, compared to 7.93 ± 7.03 days for those with susceptible strains. Statistical analysis revealed a significant difference in hospitalization duration between the two groups (*U* = 7875; *Z* = −5.64; *p* < 0.0001). Patients with MDR infections required a hospitalization period more than twice as long as non-MDR patients, reflecting the increased clinical complexity and severity associated with resistant infections.

Patients exhibiting MDR strains presented with a high burden of significant comorbidities: 157 out of 181 patients (86.7%) had cardiovascular conditions, 79 (43.6%) had neurological disorders, 88 (48.6%) experienced renal dysfunction, and 53 (29.3%) were diagnosed with diabetes mellitus. Additionally, pulmonary issues were noted in 55 patients (30.4%), neoplasms were noted in 42 (23.2%), hepatic dysfunction was noted in 22 (12.2%), HIV infection was noted in five (2.8%), and hematological disorders were noted in three (1.7%).

Among the 181 patients with MDR isolates, 112 (61.9%) were carriers of IUCs, highlighting a strong association between medical device utilization and the requirement for continuous medical intervention. Analysis of the association between specific pathogens and identified comorbidities revealed no significant correlations regarding cardiovascular, pulmonary, renal, hepatic, neurological, or diabetic conditions (all *p* > 0.05).

However, significant associations were identified between HIV status and specific pathogens (*X*^2^ = 32.36; *p* = 0.001). Notably, 40% (2/5) of HIV-positive patients were infected with *K. pneumoniae*, and 40% (2/5) harbored *Providencia* spp. These findings suggest a complex interplay between the immunological status of patients living with HIV and specific bacterial uropathogens.

Furthermore, no significant associations were detected between pathogen type and the presence of a urinary catheter (*p* > 0.05). Regarding sex-specific comorbidities, cardiovascular conditions were significantly more prevalent in female patients (*X*^2^ = 7.61; *p* = 0.001), with a prevalence of 92.5% compared to 78.4% in men. Conversely, renal comorbidities were notably more frequent among males (*X*^2^ = 11.1; *p*= 0.001), with 63.5% of men affected versus 38.3% of females. This disparity suggests that men may have a heightened predisposition to renal dysfunction in the context of bacterial infections. No significant sex-based differences were found regarding diabetes mellitus (*p* > 0.05).

The average LOH was significantly prolonged for patients managed with urinary catheters (*U* = 2386.5; *Z* = −4.32; *p* < 0.0001). This finding implies that catheterization is a marker for extended hospitalization, likely reflecting the severity of the underlying clinical conditions.

To identify independent predictors of MDR, a binary logistic regression model was developed. The variable “pathogen” was consolidated by distinguishing common isolates (*E. coli*, *K. pneumoniae*, *Proteus* spp., *Pseudomonas aeruginosa*, and *Providencia* spp.) from “rarely encountered pathogens” (*Acinetobacter baumannii*, *Morganella* spp., *Myroides* spp., *Citrobacter* spp., *Enterobacter cloacae*, *K. oxytoca*, *Serratia* spp., and *Pantoea* spp.). This consolidation was necessary to ensure model stability and avoid ambiguous interpretations caused by low-frequency isolates.

The model incorporated four predictive factors: patient age, sex, LOH, and pathogen type. The resulting model demonstrated robust statistical significance (*X*^2^ = 133.93; *p* < 0.0001), explaining between 34% and 46% of the observed variance. The Hosmer-Lemeshow test confirmed the model’s goodness-of-fit (*p* = 0.28). Quantitative evaluation revealed that each additional year of age was associated with a 3.7% increase in the probability of being infected with an MDR isolate (OR = 1.037; 95% CI: 1.7–5.8%). Furthermore, each additional day of hospitalization contributed to a 5.2% increase in the probability of being infected with an MDR isolate (OR = 1.052; 95% CI: 1.016–1.089).

Specific pathogens significantly influenced the model; infections involving *E. coli* were associated with an 89.2% reduction in the probability of having an MDR isolate (*p* < 0.001). In conclusion, patient age and LOH serve as critical predictors of having an MDR isolate, while certain pathogens, particularly *E. coli*, demonstrate a protective effect against resistance. These predictive data are summarized in Table 2.

### 3.5. Antimicrobial Susceptibility Profiles

The antimicrobial susceptibility analysis revealed significant variations across the isolated pathogens, highlighting a significant challenge in selecting effective empirical therapies. Regarding monobactams, while combinations with beta-lactamase inhibitors showed stable susceptibility, other monobactams exhibited distinct resistance patterns. Resistance was observed in 5.6% of *E. coli* strains and 34.2% (38/111) of *K. pneumoniae* isolates. Among less common pathogens, *Providencia* spp. demonstrated 66.7% susceptibility, whereas *Myroides* spp. showed a 66.7% resistance rate. In contrast, all *Morganella morganii* isolates (*n* = 2) were fully susceptible [21].

In testing for fosfomycin, *E coli* showed high susceptibility (90.2%, 129/143) while 9.8% (*n* = 14) were resistant. *K. pneumoniae* demonstrated a comparable susceptibility rate of 91.9% (102/111). Conversely, *Enterobacter cloacae* was 100% resistant, whereas *Pantoea* spp. were fully susceptible.

Nitrofurantoin susceptibility was notably high for *E. coli* (90.2%) and *Enterobacter cloacae* also showed complete susceptibility to this agent. Notably, *Enterococcus* spp. exhibited a 93.8% susceptibility to nitrofurantoin.

### 3.6. Resistance Patterns of High-Risk Pathogens

Resistance to trimethoprim-sulfamethoxazole was extensive. *Acinetobacter baumannii*, *Enterobacter cloacae*, and *Pantoea* spp. exhibited 100% resistance. High resistance rates were also documented for *K. pneumoniae* (80.2%), *Proteus* spp. (63.6%), *Providencia* spp. (66.7%), and *Pseudomonas aeruginosa* (61.5%). Only *K. oxytoca* demonstrated 100% susceptibility to this combination.

For polymyxins, often considered last-resort therapies, *Acinetobacter baumannii* remained susceptible (83.3%). *K. pneumoniae* exhibited a 27% resistance rate, while *Pseudomonas aeruginosa* showed a mixed profile with 53.8% susceptibility.

Regarding quinolones, *E. coli* showed a 14% resistance, whereas *K. pneumoniae* and *Pseudomonas aeruginosa* reached resistance levels of 44% and 69.2%, respectively. *Acinetobacter baumannii*, *E. cloacae*, and *Pantoea* spp. were entirely resistant to this class.

### 3.7. ESBL-Producing Strains and Advanced Resistance

The prevalence of ESBL-producing strains significantly complicated therapeutic options. For cephalosporins, resistance reached 27.3% for *E. coli* and a staggering 84.7% for *K. pneumoniae*. Furthermore, 55.9% of *K. pneumoniae* strains were resistant to cephalosporins combined with beta-lactamase inhibitors.

For piperacillin-tazobactam, 88.3% of *K. pneumoniae* isolates and 38.5% of *E. coli* isolates demonstrated resistance. Fifth-generation cephalosporins also showed limited efficacy, with resistance rates of 64.5% for *K. pneumoniae* and 61.5% for *Pseudomonas aeruginosa*. Additionally, carbapenems failed against 22.5% of *K. pneumoniae* and 66.7% of *Providencia* spp. isolates.

## 4. Discussion

Our results confirm that advancing age is a critical determinant of UTI prevalence, with rates increasing from 9% in adults (18–64 years) to 27.5% in those aged 85 and older. This trend highlights the escalating clinical burden within geriatric populations [24]. Notably, the female predominance in our cohort (nearly double the number of males) aligns with established anatomical and physiological vulnerabilities, including neurogenic bladder dysfunction and shorter urethral length.

Furthermore, our model quantifies the impact of aging on antimicrobial resistance, demonstrating that each additional year of age increases the probability of infection with an MDR strain by 3.7% [25,26]. This “perfect storm” for resistant strain colonization is likely driven by physiological frailty, cumulative antibiotic exposure, and frequent healthcare interactions.

Interestingly, we observed a “gender paradox” regarding severity. While females were more frequently infected, male patients faced a significantly higher risk of infection with an MDR strain (66.7%) and a longer mean LOH (16.44 vs. 9.66 days). This disparity is likely due to the inherent complexity of male UTIs, often associated with prostatic hypertrophy and biofilm formation, and a higher frequency of renal dysfunction (63.5%) in our male subgroup.

The microbiological landscape revealed a clear demarcation between departments. *E. coli* was the predominant isolate in the IDD but was entirely absent in the ICU, confirming its role as the primary agent of community-acquired UTIs. In this study, *E. coli* acted as a “protective” factor against MDR strain, as these strains typically retained higher susceptibility to first-line agents compared to hospital-adapted pathogens.

In contrast, *Acinetobacter baumannii* was found exclusively in the ICU, reinforcing its status as a quintessential nosocomial pathogen capable of surviving on abiotic surfaces and exploiting invasive procedures (e.g., mechanical ventilation) [27]. Furthermore, the emergence of *Enterococcus* spp. likely reflects the selective pressure exerted by broad-spectrum cephalosporins, which lack activity against enterococci, necessitating more nuanced empirical approach in high-dependency units.

Infection with an MDR strain more than doubled the mean LOH (17.32 vs. 7.93 days), posing a significant challenge to hospital resource management [28,29]. Beyond the difficulty of treating the primary infection, an extended stay serves as a marker for increased morbidity and the risk of secondary complications, such as *Clostridioides difficile* infections or fungal overgrowth, both of which are exacerbated by prolonged antimicrobial pressure [30].

Our findings underscore the potential of fosfomycin and nitrofurantoin as cornerstones of antimicrobial stewardship. Both agents maintained high susceptibility rates (>90%) against *E. coli*, including MDR and ESBL-producing isolates [28]. While literature suggests that a 5-day nitrofurantoin regimen may offer superior microbiological resolution compared to single-dose fosfomycin [29], both remain viable alternatives to de-escalate from carbapenems or quinolones in uncomplicated cases.

Conversely, the therapeutic window for traditional oral therapies is narrowing:

- *Acinetobacter baumannii*: Exhibited 100% resistance to TMP-SMX and quinolones [30].
- Polymyxins: While often a “last-resort” option, the 27% resistance rate observed in *K. pneumoniae* is an alarming trend that requires rigorous surveillance [31,32].
- Cephalosporins/Beta-lactamase inhibitors: We noted a dramatic decline in efficacy. In 2012, resistance rates for *K. pneumoniae* were 6.9–17.8%; our 2025 data show this has surged to 55.9% [33].

The high resistance of *K. pneumoniae* (≈90%) and *E. coli* (≈40%) to penicillin/beta-lactamase inhibitor combinations (e.g., Piperacillin-Tazobactam) is concerning [34]. To preserve their clinical utility in complicated ESBL-related UTIs (where MIC ≤ 8 mg/L), clinicians should prioritize optimized dosing strategies, such as loading doses followed by extended or continuous infusions, to maximize the time the free serum concentration remains above the MIC (T > MIC) [35].

## 5. Conclusions

The findings of this study underscore the dramatic and alarming escalation in antimicrobial resistance since 2012, particularly among ESBL-producing pathogens [28,36]. By 2025, resistance in *K. pneumoniae* strains to cephalosporin/beta-lactamase inhibitor combinations reached 55.9% while other species (such as *Myroides* spp. and *Providencia* spp.) exhibited near-total or substantial resistance [37]. Although high-dose piperacillin-tazobactam remains a viable therapeutic pathway for complicated UTIs, the observed resistance rates of 88.3% in *Klebsiella* spp. and nearly 40% in *E. coli* signal a narrowing of its clinical efficacy.

Demographic analysis identifies advancing age as a primary driver of both infection prevalence and resistance complexity, with MDR probability increasing annually. The marked predominance of female patients mirrors global trends regarding anatomical and physiological vulnerabilities. Furthermore, the strong associations between prolonged hospitalization, indwelling catheters, and specific comorbidities like HIV emphasize the need for precision-based antimicrobial stewardship. Ultimately, these trends necessitate an urgent reassessment of treatment protocols, prioritizing optimized dosing strategies, such as loading doses and continuous infusions, to combat the rising burden of highly resistant uropathogens.

## Figures and Tables

**Table 1:** Distribution of Uropathogenic Isolates by Hospital Department.

Pathogen	IDD	PD	ICU	RR	TS	Total Isolates	%
*Escherichia coli*	129	12	0	1	1	143	44.97
*Klebsiella pneumoniae*	77	13	18	3	0	111	34.91
*Enterococcus* spp.	7	8	0	1	0	16	5.03
*Pseudomonas aeruginosa*	10	2	1	0	0	13	4.09
*Proteus*	9	1	1	0	0	11	3.46
*Acinetobacter* spp.	0	0	6	0	0	6	1.89
*Providencia*	6	0	0	0	0	6	1.89
*Myroides* spp.	2	0	1	0	0	3	0.94
*Citrobacter freundii*	1	1	0	0	0	2	0.63
*Morganella morganii*	2	0	0	0	0	2	0.63
*Serratia*	2	0	0	0	0	2	0.63
*Enterobacter cloacae*	1	0	0	0	0	1	0.31
*Klebsiella oxytoca*	1	0	0	0	0	1	0.31
*Pantoea* spp.	1	0	0	0	0	1	0.31
*Total sampes/DEPT.*	248	37	27	5	1	318	100

IDD = Infectious Disease; PD = Pulmonology Department; RR = Respiratory Rehabilitation; TS = Thoracic Surgery; ICU = Intensive Care.

**Table 2:** Key Predictors of Being Infected with Multidrug-Resistant Strain.

Variables in the Equation								
	B	S.E.	Wald	df	Sig.	Exp (B)	95% C.I. for Exp (B)	
							Lower	Upper
sex	−0.474	0.354	1.796	1	0.180	0.623	0.311	1.245
age	0.036	0.010	13.134	1	0.000	1.037	1.017	1.058
days of hospitalization	0.050	0.018	8.183	1	0.004	1.052	1.016	1.089
pathogen			56.141	6	0.000			
*E. coli*	−2.221	0.740	9.019	1	0.003	0.108	0.025	0.462
*Klebsiella pneumoniae*	0.635	0.757	0.705	1	0.401	1.888	0.429	8.317
*Proteus* spp.	−1.639	0.947	2.998	1	0.083	0.194	0.030	1.242
*Pseudomonas aeruginosa*	−0.970	0.910	1.137	1	0.286	0.379	0.064	2.255
*Providencia* spp.	−0.251	1.348	0.035	1	0.853	0.778	0.055	10.928
Constant	−1.432	1.018	1.980	1	0.159	0.239		

Pathogen = categorical variable representing uropathogen species; its inclusion significantly enhances the model’s predictive power.

## Data Availability

The data presented in this study are available on request from the corresponding author (due to internal regulations of the hospital—Regulation UE nr. 679 from 2016 regarding protection of personal data).

## Abbreviations

The following abbreviations are used in this manuscript:

AMR	Antimicrobial resistance
AST	Antimicrobial susceptibility test
BSI	Bloodstream infection
CAUTI	Catheter-associated urinary tract infection
CDC	Centers for Disease Control and Prevention
CI	Confidence Interval
CKD	Chronic kidney disease
CFU	Colony-forming units
CRE	Carbapenem-resistant Enterobacteriaceae
*E. coli*	*Escherichia coli*
ECDC	European Centre for Disease Prevention and Control
ESBL	Extended-spectrum beta-lactamase
GNB	Gram-negative bacteria
HIV	Human Immunodeficiency Virus
ICU	Intensive Care Unit
IDD	Infectious disease department
IUC	Indwelling urinary catheter
*K. oxytoca*	*Klebsiella oxytoca*
*K. pneumoniae*	*Klebsiella pneumoniae*
LOH	Length of hospital stay
MDR	Multi-drug resistance
MIC	Minimum inhibitory concentration
OR	Odd ratio
PD	Pulmonology Department
*P. mirabilis*	*Proteus mirabilis*
spp.	Species
RRD	Respiratory Rehabilitation Department
TMP-SMX	trimetoprim-sulfamethoxazol
UTI	Urinary tract infection
